# Ligand-Doped Copper Oxo-hydroxide Nanoparticles are Effective Antimicrobials

**DOI:** 10.1186/s11671-018-2520-7

**Published:** 2018-04-19

**Authors:** Carlos A. P. Bastos, Nuno Faria, Angela Ivask, Olesja M. Bondarenko, Anne Kahru, Jonathan Powell

**Affiliations:** 1 Biomineral Research Group, Department of Veterinary Medicine, University of Cambridge, Madingley Road, Cambridge, CB3 0ES UK; 2 Biomineral Research Group, Department of Mineral Science and Technology, MRC Elsie Widdowson Laboratory, Fulbourn Road, Cambridge, CB1 9NL UK; 3 National Institute of Chemical Physics and Biophysics, Laboratory of Environmental Toxicology, Akadeemia tee 23, 12618 Tallinn, Estonia; 4 Estonian Academy of Sciences, Kohtu 6, 10130 Tallinn, Estonia

**Keywords:** Copper, Nanoparticle, Antibacterial activity, Topical antimicrobial, Bacterial biosensor, Labile nanoparticle

## Abstract

Bacterial resistance to antimicrobial therapies is an increasing clinical problem. This is as true for topical applications as it is for systemic therapy. Topically, copper ions may be effective and cheap antimicrobials that act through multiple pathways thereby limiting opportunities to bacteria for resistance. However, the chemistry of copper does not lend itself to facile formulations that will readily release copper ions at biologically compatible pHs. Here, we have developed nanoparticulate copper hydroxide adipate tartrate (CHAT) as a cheap, safe, and readily synthesised material that should enable antimicrobial copper ion release in an infected wound environment.

First, we synthesised CHAT and showed that this had disperse aquated particle sizes of 2–5 nm and a mean zeta potential of − 40 mV. Next, when diluted into bacterial medium, CHAT demonstrated similar efficacy to copper chloride against *Escherichia coli* and *Staphylococcus aureus*, with dose-dependent activity occurring mostly around 12.5–50 mg/L of copper. Indeed, at these levels, CHAT very rapidly dissolved and, as confirmed by a bacterial copper biosensor, showed identical intracellular loading to copper ions derived from copper chloride. However, when formulated at 250 mg/L in a topically applied matrix, namely hydroxyethyl cellulose, the benefit of CHAT over copper chloride was apparent. The former yielded rapid sustained release of copper within the bactericidal range, but the copper chloride, which formed insoluble precipitates at such concentration and pH, achieved a maximum release of 10 ± 7 mg/L copper by 24 h.

We provide a practical formulation for topical copper-based antimicrobial therapy. Further studies, especially in vivo, are merited.

**Electronic supplementary material:**

The online version of this article (10.1186/s11671-018-2520-7) contains supplementary material, which is available to authorized users.

## Background

Microbial infections contribute to millions of deaths globally [1]. Often, the inefficiency of an antimicrobial treatment is due to microbial resistance to conventional antibiotics [2–5]. As such, novel antimicrobials are eagerly sought. Copper has long been recognised for its antimicrobial effects and may have the potential for greater clinical longevity than standard antibiotics since it appears to act via a multiplicity of mechanisms against bacteria, including interaction with bacterial proteins and DNA, production of reactive oxygen species (ROS), and disruption of membrane integrity [6, 7]. For the same reason, it is suggested that the potential for antimicrobial resistance of pathogenic bacterial strains to copper and other metals is limited [7–9]. In addition, copper is relatively cheap and is of low toxicity to humans since its essentiality at trace levels has ensured the evolution of tight homeostatic control [10–12]. There is, therefore, common use of this metal for preventative infection measures, mostly to avoid bacterial biofilm formation on surfaces in high-risk areas such as hospitals and nursing homes [13, 14]. In contrast, copper has not found significant therapeutic use in topical antimicrobial formulations, unlike silver which is widely employed [15].

Bacteria are susceptible to copper loading in their intracellular environment, and the effectiveness of a copper source is related to its ability to release copper ions [16, 17]. In this respect, a significant challenge for copper-based antimicrobials is the achievement of a concentrated formulation that allows the sustained release of antimicrobial copper at effective concentrations into fluids such as wound exudate. This is because copper is a hydrolytic metal ion and as its concentration is increased at the pH of typical topical formulations (i.e. near neutral), so does its tendency to induce hydrolysis and form insoluble oxo-hydroxides [18]. At physiological pHs, these oxo-hydroxides are not good substrates for the release of soluble or, therefore, potentially efficacious, copper ions [16, 19, 20].

Recently, with the purpose of finding a bioavailable iron supplement, the issue of effective release of ferric ions from a concentrated oxo-hydroxide source under physiological conditions was solved through structural modification of the primary particles. In that work, iron was precipitated in the presence of crystal-doping GRAS ligands, namely adipic and tartaric acids, to purposefully destabilise the final ferric oxo-hydroxide structure. This strategy had the advantage of (a) preventing irreversible agglomeration of the ferric oxo-hydroxide particles and (b) greatly increasing their lability (ease of solubility) under appropriate physiological conditions. This material has been termed “iron [oxo-]hydroxide adipate tartrate” or IHAT [21, 22]. By analogy, we considered here whether copper [oxo-]hydroxide adipate tartrate (CHAT) could be synthesised and formulated at high concentrations but still release copper ions at effective antimicrobial levels. In particular, the aim of this work was to develop a cheap and scalable synthetic process that produces copper oxo-hydroxide nanoparticles that, unlike previously reported materials, should readily release biocidal concentrations of copper ions in a simulated wound environment.

Thus, in this study, we synthesised CHAT and characterised its ability to deliver bioavailable copper and, hence, to demonstrate antimicrobial activity. We have concentrated on strains of *Escherichia coli* as “indicator” species for Gram-negative bacteria [19, 23] but have additionally demonstrated proof-of-principle effects against *Staphylococcus aureus*, as Gram-positive bacteria that often obtain multidrug resistance. Hence, the study aimed to assess the value of developing CHAT further for clinical applications in topical antimicrobial therapy.

## Methods

Unless otherwise stated, all experiments were performed using ultra-high purity (UHP) water (reverse osmosis purification; 18.2 ΩM/cm), at room temperature (20 ± 2 °C), and all reagents were purchased from Sigma Aldrich.

### Copper Formulations and CHAT Nanoparticles

Copper chloride stocks (40 mM copper) were prepared by dissolving CuCl_2_·2H_2_O in water. Copper oxide nanoparticle (CuO NPs; Sigma 544868) stocks were prepared from a commercial powder that was free of impurities, had a primary particle size of 34 nm (range 10–50 nm), and was previously tested as an antimicrobial agent [24–26]. These stocks were prepared at 1.3 g/L copper by dispersing the powder in water with vigorous agitation. Colloidal suspensions of CHAT nanoparticles were synthesised using a co-precipitation method [27]. Briefly, copper chloride, tartaric acid, and adipic acid were dissolved in water to achieve a molar ratio of copper/tartaric acid/adipic acid in the final suspension of 2:1:1 and a copper concentration of 2.5 g/L. The initial pH of the mixture was always below 2.5, and the copper was fully solubilised. The pH was then slowly increased by a drop-wise addition of a concentrated solution of NaOH (5 M) with constant agitation until pH 8.2 ± 0.2.

### Copper Content and Phase Distribution of CHAT Suspensions

Copper content in colloidal suspensions was determined by inductively coupled plasma-optical emission spectrometry (ICP-OES, Jobin Yvon 2000, Horiba). All samples were diluted down to concentrations below 100 mg/L in 5% HNO_3_ (*v*/*v*) at least 24 h prior to analysis to ensure full solubility of copper. Calibration standards (0.1 to 100 mg/L copper) were matrix-matched in 5% HNO_3_, and copper quantification was carried out at 324.754 nm. Fractionation of the copper into percentages of agglomerated, nanoparticulate, and soluble copper was achieved by filtration and ultrafiltration of CHAT stocks. Suspensions were filtered (200 nm cut-off), and the retentate was considered as the agglomerated fraction. In order to isolate the soluble copper and to distinguish it from nanoparticulate copper, the colloidal suspension was ultrafiltered through a 3-KDa filter (Sartorius Vivaspin 500 VS0192; 16,000×*g*, 5 min) as this corresponds to a cut-off below 1 nm (Zetasizer Software 7.11, Malvern Instruments Ltd). The copper content of all fractions (total, 200 nm filtrate, 3 KDa ultrafiltrate) was determined by ICP-OES, and fractions expressed as percentage in relation to total copper content are as follows:

$$ {\displaystyle \begin{array}{l}\%\mathrm{Soluble}\ \mathrm{Copper}\ \left(<1\mathrm{nm}\right)\%\kern0.5em =\frac{\kern0.5em {Cu}_{3\mathrm{KDa}}}{Cu_{\mathrm{Total}}}\times 100\\ {}\%\mathrm{Agglomerated}\ \mathrm{Copper}=\frac{\ {Cu}_{\mathrm{Total}}-{Cu}_{<200\mathrm{nm}}\kern0.5em }{Cu_{\mathrm{Total}}}\kern0.5em \times 100\\ {}\%\mathrm{Nanoparticulate}\kern0.5em \mathrm{Copper}\kern0.5em =100-\%\mathrm{Agglomerated}\ \mathrm{copper}-\%\mathrm{Soluble}\ \mathrm{copper}\end{array}} $$

### Determination of Copper Content and Copper to Ligand Ratios in Dry CHAT Nanoparticles

CHAT nanoparticles were agglomerated and precipitated to enable recovery and removal of unbound components. To enable this, ethanol was added to colloidal suspensions of CHAT (2.5 g/L copper) at a ratio of 2:1 ethanol/suspension (*v*/*v*), and the resulting CHAT agglomerates were recovered by centrifugation (4500×*g* × 15 min in a Mistral 6000). The solution phase, containing unbound ligand species, was discarded. Determination of copper content in solid-phase CHAT was as follows. A powder was produced by oven-drying the ethanolic precipitated pellet to constant weight at 45 °C. This was then milled and 35.2 ± 0.3 mg (*n* = 2) was digested in 11 ± 1 g of 70% HNO_3_, with accurate weights recorded. Once fully digested, this solution was diluted 20-fold in water and the copper concentration determined by ICP-OES. Ligand to copper ratios were determined directly from dried, ethanol-precipitated CHAT agglomerates. The agglomerates were first re-suspended in water to their original volume to facilitate dissolution with lower amounts of HCl—a requirement for high-performance liquid chromatography (HPLC) analysis. Aliquots were either dissolved in 5% HNO_3_ for ICP-OES analysis of copper (as described above) or in 80 mM HCl for HPLC analysis of ligands (tartaric and adipic acids). Ligand analysis was carried out in a standard reverse phase chromatography system (C18 column in a Waters Alliance 2690/5 equipped with a 2998 PDA detector; further details are given in Additional file 1).

### Physicochemical Characterisation of CHAT Suspensions

Hydrodynamic particle size distribution was determined by dynamic light scattering (DLS; Zetasizer NanoZS, Malvern Instruments Ltd). Aliquots of CHAT colloidal suspensions (2.5 g/L copper) were transferred to a 1-mL disposable cuvette, and measurements (*n* = 3) were carried out at 25 ± 2 °C. Again, the exact settings are shown in Additional file 2. The zeta potential of CHAT suspensions was determined by laser Doppler micro-electrophoresis (Zetasizer NanoZS, Malvern Instruments Ltd) using disposable folded capillary cells (DTS1070) and assuming a dielectric constant of 78.5 and a viscosity of 0.89 cP. Transmission electron microscopy (TEM) characterisation was carried out applying a droplet of CHAT suspension to holey carbon perforated grids and drying at 50 °C overnight. Grids were then imaged on the TEM (FEI-Philips CM100) at 120 kV in bright-field mode.

### Antimicrobial Activity of Copper Formulations

Assays were carried out in heavy metal MOPS (HMM) medium, a recognised metal-ion compatible medium (Additional file 3), which was supplemented with 0.4% glucose and 0.1% casein acid hydrolysate, and pH adjusted to 7.2 ± 0.2 [28]. Prior to the addition of copper compounds, *Escherichia coli* (NCTC11100) and *Staphylococcus aureus* RN4220 [29] were grown overnight at 30 °C under constant shaking in an Infors HT Minitron incubator at 80 rpm. Afterwards, the bacterial suspensions were diluted to an optical density of 0.05–0.1 (ca. 10^6^ cells/mL) at 595 nm for *E. coli (*Multiskan RC 351 Labsystem) or 600 nm for *S. aureus* (Multiskan plate reader, ThermoFisher Scientific). Next, stocks of copper chloride and colloidal CHAT were diluted in HMM and added to the bacterial suspensions to obtain final copper concentrations between 0.4 and 100 mg/L. Incubation then took place for a period of 6 to 9 h, and bacterial growth was determined by monitoring optical density as a measure of bacterial biomass.

Copper solubility over time in bacterial growth medium was determined by diluting copper chloride and colloidal CHAT stocks in HMM to 12.5, 25, and 50 mg/L copper and determining the fraction of soluble copper at 0, 2, 4, and 8 h through ultrafiltration (3 KDa) and ICP-OES analysis, as described above.

### Intracellular Bioavailability of Copper Formulations

Recombinant bioluminescent Cu-sensing bacteria, *E. coli* MC1061 (pSLcueR/pDNPcopAlux), which respond to sub-toxic amounts of bioavailable copper by increasing their bioluminescence were used to quantify the bioavailability of copper compounds [30]. Bacterial suspensions were prepared as described for the antimicrobial activity assay and incubated with a series of dilutions of copper chloride and CHAT (0 to 50 mg/L copper) on 96-well microplates for 4 h. Bioluminescence was measured with an Orion II Plate Luminometer (Berthold Detection Systems), and induction of bioluminescence was calculated as follows:

$$ Bioluminescence\ in duction, Fold\ Change=\frac{ Bioluminescen ce\ in\  Cu\  exposure}{Bioluminescen\mathrm{ce}\  without\  Cu\ } $$

### Intracellular Stress Induced by Copper Formulations

The ability of copper compounds to induce intracellular superoxide anions and single-strand DNA breaks was assessed with recombinant bioluminescent bacteria, *E. coli* K12::soxRSsodAlux and *E. coli* MC1061 (pDEWrecAlux), respectively [17]. Bacterial cultures were prepared as described for the antimicrobial assay, and bacteria were exposed to a series of dilutions of copper chloride and CHAT (0 to 50 mg/L copper) on white 96-well microplates over 4 h. The biosensors’ performances were controlled by exposing bacteria to the superoxide anion-inducing chemical menadione (0.04–30 μg/L), or to hydrogen peroxide (0.1–150 mg/L), as positive controls for *E. coli* K12::soxRSsodAlux or *E. coli* MC1061 (pDEWrecAlux), respectively. Again, bacteria were incubated on white 96-well microplates and bioluminescence was measured with an Orion II Plate Luminometer and induction of bioluminescence was calculated as in Eq. 5.

### Incorporation of Copper Formulations in Hydroxyethyl Cellulose Gels

Stocks of copper chloride, CHAT, and commercial, unmodified copper oxide nanoparticles (CuO NPs) were diluted in UHP water to 250 mg/L copper. The resulting suspensions of CHAT and CuO NPs were at near neutral pH and could be incorporated directly in the gel, but copper chloride solution was still acidic after dilution and was therefore adjusted to pH 7.0 ± 0.2. Hydroxyethylcellulose (HEC) was then dissolved directly (2% *w*/*v*) into the various diluted stocks using a roller mixer (Denley Spiramix 5) until homogenous gels were formed. Ten grams of each gel was transferred into Falcon tubes and allowed to settle overnight. Next, 10 mL of freshly prepared 50 mM sodium bicarbonate buffer (dissolved from NaHCO_3_ powder and adjusted to pH 7.0 ± 0.2) was transferred into each tube with care to minimise disturbances at the gel-liquid interface (specific surface area of 7.1 cm^2^). Aliquots were then collected and analysed by ICP-OES to determine copper release over time.

## Results

**Fig. 1:**
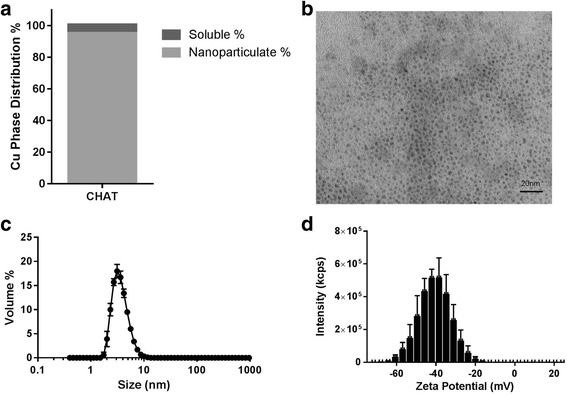
Characterisation of CHAT stock solution. **a** Copper phase distribution at 2.5 g/L CHAT: soluble (< 3 KDa) and nanoparticulate percentage. **b** Nanoparticle dispersion imaging by TEM. **c** Hydrodynamic particle size distribution of freshly prepared particles, as determined by dynamic light scattering. **d** Zeta potential distribution (*n* = 3; error bars represent standard deviations)

As described in the “Methods” section, CHAT was synthesised in a similar fashion to its iron analogue, IHAT [21, 22], by doping copper oxo-hydroxide (2.5 g/L copper) with tartaric and adipic acids. This produced stable colloidal suspensions in which all copper went through a 200-nm filter but very little (5%) passed a 3-KDa filter. This indicated that most copper was nanoparticulate (95%; Fig. 1a) with little “free” copper and no detectable large agglomerates—again like the IHAT analogue [21, 22]. When precipitated in ethanol, to remove unbound ligand species, and then dried, CHAT contained 31 ± 1% copper (*w*/*w*) by ICP-OES analysis. Copper to ligand molar ratios, the latter determined by HPLC, were 2:1 for copper to tartrate and 2:0.3 for copper to adipate. CHAT particles appeared almost monodisperse with diameters of 2–3 nm by TEM imaging (Fig. 1b). These findings were consistent with hydrodynamic sizing data for CHAT plus a hydration shell since the median diameter by volume in UHP water was 3.4 nm (Fig. 1c) and the size distribution was narrow (2.4–5.6 nm for 80% of the volume) when assessed with dynamic light scattering. The average zeta potential was − 39 mV (Fig. 1d), consistent with the nanoparticles that form a stable aquated dispersion [27], and, indeed, the CHAT stock suspension was shown to be stable for several years (Additional file 4).

**Fig. 2:**
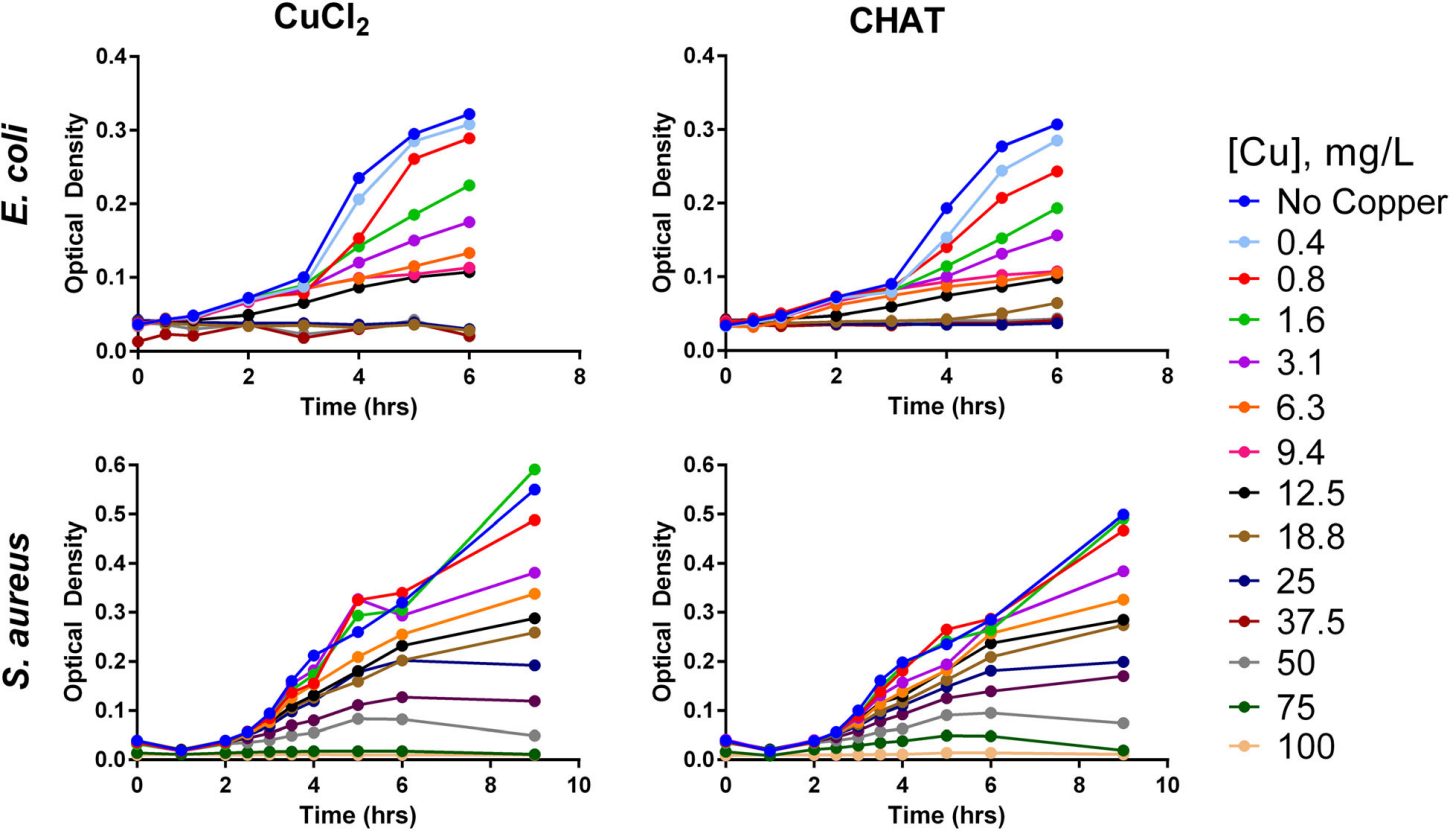
*Escherichia coli* (top) and *Staphylococcus aureus* (bottom) growth curves, represented here as optical density, upon exposure to different concentrations of copper chloride (left) or CHAT (right) in supplemented HMM.

Next, we considered the antimicrobial activity of CHAT when stock suspensions were diluted in bacterial growth media to concentrations associated with the antimicrobial activity of copper salts. For CHAT and copper chloride, the growth inhibition curve was very similar for both *E. coli* and *S. aureus* with most activity occurring at total copper concentrations between the 12.5 and 50 mg/L range (Fig. 2). Complete *E. coli* growth inhibition was observed upon incubation with 18.8 (CuCl_2_) and 25 (CHAT) mg/L copper, whilst for *S. aureus*, full growth inhibition was obtained at 75 (CuCl_2_) and 100 (CHAT) mg/L copper (Fig. 2; Percentage growth inhibition vs copper concentration is provided in Additional file 5).

**Fig. 3:**
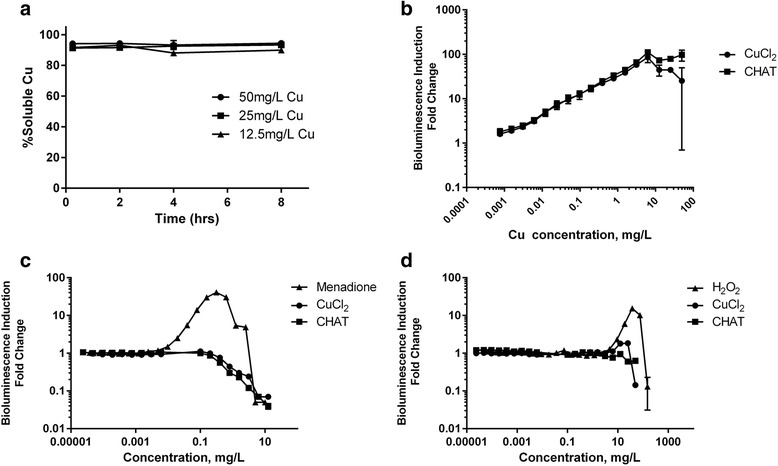
**a** Dissolution profile of CHAT in supplemented HMM at 12.5, 25, and 50 mg/L copper. Dose-response of bioluminescence induction of recombinant luminescent bacteria: **b** intracellular copper ion-responding *E. coli* MC1061 pSLcueR/pDNPcopAlux bacteria, **c** DNA damage-responding *E. coli* MC1061 (pDEWrecAlux), and **d** superoxide anion-responding *E. coli* K12::soxRSsodAlux upon exposure for 4 h in supplemented HMM to copper chloride, CHAT (concentration in mg Cu/L), and respective controls (menadione in **c** and H_2_O_2_ in **d**)

Indeed, at these antimicrobial concentrations, at least 94% of CHAT was rapidly solubilised (within 15 min), again as judged by ultrafiltration and ICP-OES analysis (Fig. 3a). We therefore anticipated that the antimicrobial effectiveness of CHAT was linked to this chemical lability, with rapid dissolution of the nanoparticles allowing intracellular bacterial acquisition of copper ions. To test this, we challenged the Cu-sensing *E. coli*, MC1061 (pSLcueR/pDNPcopAlux), in which bioluminescence increases in response to sub-toxic concentrations of intracellular copper ions [30], with 0 to 50 mg/L copper as CHAT or copper chloride. Increasing concentrations in the culture medium of both sources of copper led to increasing bioluminescence in the *E. coli* copper sensor strain (Fig. 3b), consistent with rises in intracellular copper. The slope of the dose-response curve was identical up to 6.25 mg/L for both sources of copper confirming that bioavailable copper from CHAT was comparable to a fully solubilised source. Thereafter, at concentrations up to 50 mg/L copper, the luminescence did not increase due to the toxicity of both copper compounds (Fig. 3b).

In parallel to studying intracellular copper in *E. coli* exposed to solutions prepared with CHAT or copper chloride, we also tested the ability of these solutions to trigger intracellular superoxide anions or to cause bacterial DNA damage in different *E. coli*-based biosensors. In neither case was there a significantly observable effect, despite the sensors responding to relevant positive controls, namely hydrogen peroxide and menadione, respectively (Fig. 3c, d). Taken together, the equivalent responses of the three bacterial biosensors to solutions prepared from different chemical forms of copper strongly support the notion that, in both cases, bacteria were being exposed to the same soluble copper, in spite of one formulation starting out as nanoparticulate.

**Fig. 4:**
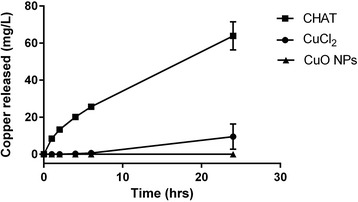
Copper release from HEC matrices containing CHAT, copper chloride, or copper oxide nanoparticles (CuO NPs), all at 250 mg/L copper

Finally, as noted above, the advantage of CHAT over soluble copper salts would only be apparent if a concentrated formulation allowed the former to retain its chemical lability unlike the latter. Using hydroxyethyl cellulose (HEC), a common aqueous base for topical formulations [31–33], we incorporated 250 mg/L copper as either copper chloride, CHAT, or as the commercial CuO NPs. When 10 mL of 50 mM NaHCO_3_ buffer, as a simplified wound exudate, were added to 10 g of each of the copper-incorporated HEC gels (i.e. 2.5 mg copper), there was sustained release of copper from the CHAT-containing preparation, to greater than 60 mg/L by 24 h (Fig. 4). Moreover, release was relatively rapid with antimicrobially active concentrations being achieved by 2–4 h. In contrast, pH-neutralised copper chloride was a poor substrate for copper release, as anticipated by its tendency to hydrolyse and form agglomerates of copper oxo-hydroxides, so by 24 h, only 10 ± 7 mg/L copper had been achieved in solution (Fig. 4). The commercial CuO NPs yielded no discernible copper release at all (Fig. 4).

## Discussion

We show here that a copper-based nanomaterial, namely CHAT, can be formulated at high concentrations, unlike previously described copper-based nanoparticles [34, 35], whilst retaining its properties as a labile source of bioavailable copper with antimicrobial efficacy. As noted above, the synthesis of CHAT was inspired following many years’ previous work on the iron analogue, IHAT [21, 22]. This, in turn, was inspired by nature’s solution to rapid mineral turnover in vivo, for the efficient recycling of essential metal ions, whereby organic molecules are used to destabilise the crystal structure of primary mineral particles [21, 22]. In the synthetic versions, GRAS ligands are incorporated into metal oxo-hydroxides as they form in solution from cross-linking polymers [21, 22]. Through structural destabilisation, this ensures lability of the final mineral phase and also generates highly negative nanoparticles—as demonstrated by the zeta potential measurement—which repel agglomeration and aggregation, thus producing nanoparticle suspensions that were stable for years. Here, and as previously shown for IHAT, tartrate was the dominant ligand in achieving these physicochemical changes to the copper oxo-hydroxide structure since its incorporation was ca. 3-fold greater than that of adipate—the latter behaving more so as a buffer during synthesis [21, 22].

In the absence of modification, freshly precipitated metal oxo-hydroxides will agglomerate and aggregate and will start to age, whereby they condense and gradually increase their crystallinity. These size and mineral phase transitions reduce the ability of the structures to participate in the reverse reaction, i.e. to re-dissolve. It was therefore unsurprising that when copper oxo-hydroxide was freshly formed, from pH neutralisation of a copper chloride solution, at least some soluble copper was released in our gel release assay (Fig. 4), whereas for the commercial CuO NPs, which were agglomerated and comprised a more condensed mineral phase (i.e. copper oxide), undetectable copper was released. The lack of dissolution from commercial 30 nm nanoparticles—which, regardless of their aggregation state, would have presented a large surface area for dissolution—shows that mineral phase is a key driver in the release of copper ions and that, as noted above, modification of the mineral’s primary particles, achieved here through ligand doping, is really required to bring about a marked shift in dissolution characteristics. Furthermore, the synthesis of CHAT was carried out at room temperature, since high synthetic temperature favours less amorphous phases which consequently may reduce dissolution rates. Also, room temperature synthesis has the benefit of reducing energetic costs when manufacturing at scale.

Whilst there may be other ways to formulate high, stable concentrations of copper that enables sustained release and rapid dissolution of ions when required, we cannot envisage another synthesis that is so straightforward and the cost of goods (for reactants) so low. These are important factors as the issue of topical infections and bacterial resistance are by no means restricted to developed countries. Developing countries are increasingly plagued by bacterial resistance issues and so affordable efficacious solutions are urgently required [36, 37]. Although there are insufficient studies to reach concrete solutions, there is evidence that resistance to toxic metal ions is more difficult for bacteria to achieve than resistance to conventional antibiotics [7]. The theory is predicated on the idea that copper and silver probably do not have discrete pathways of antimicrobial activity but, rather, can impact multiple targets including various enzyme systems and thus can destabilise the overall bacterial cell structure [17, 19, 38]. Indeed, it has been shown that bacteria remain susceptible to copper and certain other metals ions in spite of exposures over centuries [6, 7, 39]. Interestingly, there is recent evidence that metal-based antimicrobials can even return bacterial sensitivity to conventional antibiotics in spite of prior resistance [40, 41].

## Conclusions

Here, we have demonstrated that the issue of bioavailable copper ions, at physiological pHs and high concentrations, can be solved by doping a copper nanomineral with organic acids, in a similar strategy to that previously utilised for iron analogues [21, 22]. These copper-based nanoparticles (termed CHAT) readily dissolved in the bacterial medium, showing equivalent intracellular copper uptake and antibacterial activity to soluble copper salts. Critically, however, and unlike for simple copper salts, CHAT can be concentrated in a pH-neutral formulation and retain its lability in terms of copper ion release. Indeed, CHAT released copper ions within the bactericidal range and could thus be the basis for a novel, topical antimicrobial agent either alone or enhancing the efficacy of resisting antibiotics. With increasing antibiotic resistance, new topical antimicrobials are needed and CHAT is inexpensive, readily synthesised, and uses components that are generally recognised as safe (GRAS). In vivo studies are merited.

## Additional files

Additional file 1: HPLC measurement settings. (PDF 621 kb)

Additional file 2: Particle size measurements settings. (PDF 617 kb)

Additional file 3: Composition and preparation of Heavy Metal MOPS (HMM) medium. (PDF 638 kb)

Additional file 4: Particle size stability of aquated CHAT between 0 and 2 years. (PDF 594 kb)

Additional file 5: Bacterial growth inhibition upon incubation with CuCl2 and CHAT. (PDF 597 kb)

## Abbreviations

CHAT: Copper [oxo]-hydroxide adipate tartrate nanoparticles
CuO NPs: Copper oxide nanoparticles
DLS: Dynamic light scattering
*Escherichia coli*: *E. coli*
GRAS: Generally recognised as safe
HEC: Hydroxyethylcellulose
HMM: Heavy metal MOPS medium
HPLC: High-performance liquid chromatography
ICP-OES: Inductively coupled plasma-optical emission spectrometry
IHAT: Iron [oxo]-hydroxide adipate tartrate nanoparticles
MOPS: 3-(*N*-morpholino) propanesulfonic acid
*Staphylococcus aureus*: *S. aureus*
UHP: Ultra-high purity
