# Supplementary material for: Ligand-Doped Copper Oxo-hydroxide Nanoparticles are Effective Antimicrobials
Source: Nanoscale Res Lett. 2018 Apr 19;13:111. doi: 10.1186/s11671-018-2520-7 (PMC5908776; doi:10.1186/s11671-018-2520-7)
Supplement: Supplementary file 1 — HPLC measurement settings. (PDF 621 kb) [file 11671_2018_2520_MOESM1_ESM.pdf]

**Additional file 1. HPLC measurement settings**

Hardware
HPLC Waters Alliance 2690/5
Detector Waters 2998 PDA

Column: Purospher Star, RP-18, endcapped, 5 µm, 150 x 4.6 mm ID
Flow rate: 0.5 mL/min
Wavelength: 210 nm
Injection volume: 20 µL
Sample temperature 10 C
Column temperature 30 C

Mobile phase: A = 0.01mKH2PO4 buffer at pH 1.6, B = acetonitrile

Gradient program;

| | | | | |
|---|---|---|---|---|
| Time | Flow rate (mL | | | |
| /min) | %A | %B | Curve | |
| _ | 0.5 | 98 | 2 | _ |
| 5.00 | 0.5 | 98 | 2 | 6 |
| 6.00 | 0.5 | 85 | 15 | 6 |
| 16.00 | 0.5 | 85 | 15 | 6 |
| 17.00 | 0.5 | 98 | 2 | 6 |

Where 6 is a linear change between two sets of conditions
PDA detector conditions
Range: 200 to 400 nm
Detection: 210 nm
Sampling rate: 10 points per second
Resolution: 1.2 nm
Filter time constant: Normal.

Software: Empower 3, Waters Corp, Service release 2
