# Supplementary material for: Ligand-Doped Copper Oxo-hydroxide Nanoparticles are Effective Antimicrobials
Source: Nanoscale Res Lett. 2018 Apr 19;13:111. doi: 10.1186/s11671-018-2520-7 (PMC5908776; doi:10.1186/s11671-018-2520-7)
Supplement: Supplementary file 2 — Particle size measurements settings. (PDF 617 kb) [file 11671_2018_2520_MOESM2_ESM.pdf]

Additional file 2. Particle size measurements settings

Particle size measurements were performed using a backscattering angle of 173˚ and the following settings were set:

Material Refractive Index	0.192
Absorption	0.1
Dispersant Refractive Index	1.330
Viscosity	1.00331 mPa.s
