# Supplementary material for: Ligand-Doped Copper Oxo-hydroxide Nanoparticles are Effective Antimicrobials
Source: Nanoscale Res Lett. 2018 Apr 19;13:111. doi: 10.1186/s11671-018-2520-7 (PMC5908776; doi:10.1186/s11671-018-2520-7)
Supplement: Supplementary file 3 — Composition and preparation of Heavy Metal MOPS (HMM) medium. (PDF 638 kb) [file 11671_2018_2520_MOESM3_ESM.pdf]

Additional file 3. Composition and preparation of Heavy Metal MOPS (HMM) medium

HMM was prepared from concentrated stock solutions of each reagent, and pH adjusted to 7.2 ± 0.2 (Table 1). Freshly prepared medium was immediately autoclaved at 121°C for 15 minutes, let cool down and stored at 4 ± 2˚C. Autoclaved medium was used within a month of preparation.

Composition of HMM medium.

**Reagent**	**Concentration in HMM medium**
3-(N-morpholino)propanesulfonic acid (MOPS)	40 mM
KCl	50 mM
NH_4Cl	10 mM
$MgSO_4$	0.5 mM
$FeCl_3.6H_2O$	1 µM
Glycerol-2-Phosphate	1 mM
Glucose	0.4% (w/v)
Casein acid hydrolysate	0.1% (w/v)
