# Supplementary material for: Ligand-Doped Copper Oxo-hydroxide Nanoparticles are Effective Antimicrobials
Source: Nanoscale Res Lett. 2018 Apr 19;13:111. doi: 10.1186/s11671-018-2520-7 (PMC5908776; doi:10.1186/s11671-018-2520-7)
Supplement: Supplementary file 4 — Particle size stability of aquated CHAT between 0 and 2 years. (PDF 594 kb) [file 11671_2018_2520_MOESM4_ESM.pdf]

Additional 4. Particle size stability of aquated CHAT between 0 and 2 years

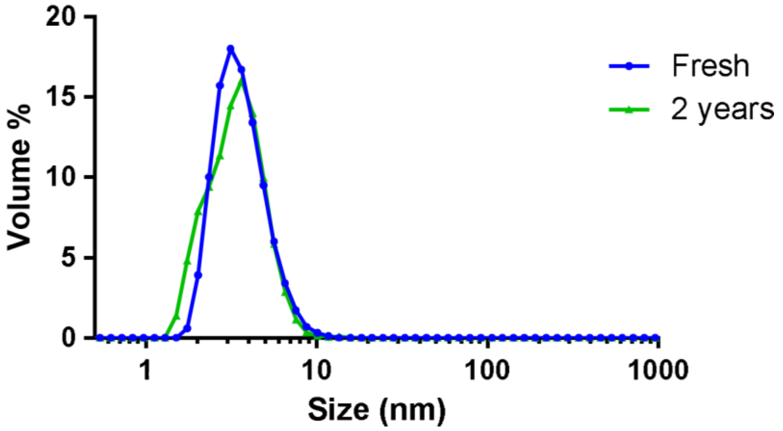

Figure S1. Hydrodynamic particle size of 40mM CHAT in water (as per conditions of synthesis), when freshly prepared and when 2 years old by Dynamic Light Scattering.
