# Supplementary material for: Ligand-Doped Copper Oxo-hydroxide Nanoparticles are Effective Antimicrobials
Source: Nanoscale Res Lett. 2018 Apr 19;13:111. doi: 10.1186/s11671-018-2520-7 (PMC5908776; doi:10.1186/s11671-018-2520-7)
Supplement: Supplementary file 5 — Bacterial growth inhibition upon incubation with CuCl2 and CHAT. (PDF 597 kb) [file 11671_2018_2520_MOESM5_ESM.pdf]

Additional file 5. Bacterial growth inhibition upon incubation with $CuCl_2$ and CHAT.

Growth inhibition was obtained by comparing copper treated and untreated bacteria, as below:

$$\text{Growth Inhibition \%} = \left(\frac{OD\ untreated - OD\ Copper\ Treated}{OD\ untreated}\right) \times 100$$

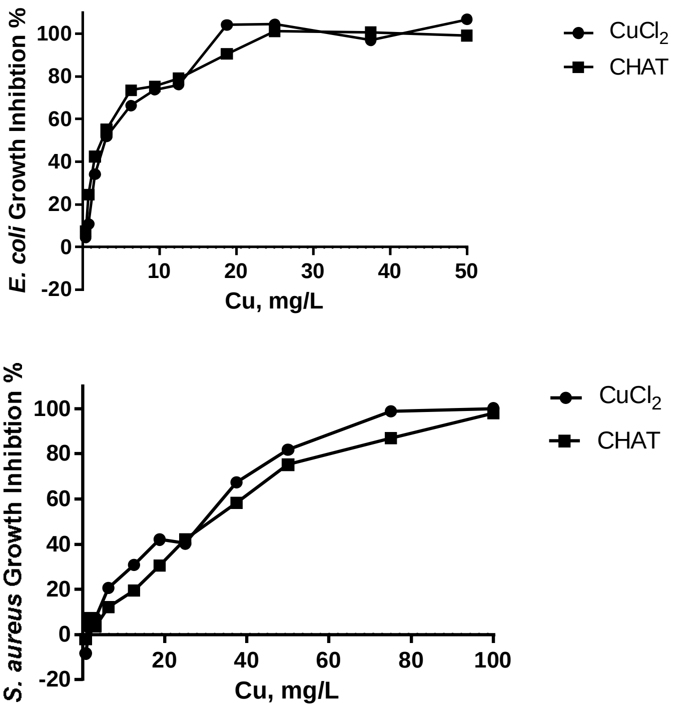

Figure S2. *E. coli* (top) and *S. aureus* (bottom) growth inhibition percentage upon incubation with several concentrations of $CuCl_2$ and CHAT in supplemented HMM for 6 hours and 9 hours, respectively.
